# Preoperative transcatheter arterial chemotherapy may suppress oxidative stress in hepatocellular carcinoma cells and reduce the risk of short-term relapse

**DOI:** 10.18632/oncotarget.17660

**Published:** 2017-05-07

**Authors:** Hao Su, Guangzhi Zhu, Ketut Indra Djaja P, Yi Lin, Yizhen Gong, Xiaoguang Liu, Jiaquan Li, Zhiming Liu, Xiao Qin, Lequn Li, Tangwei Liu, Zili Lu, Minyi Wei, Lunan Yan, Cheryl Ann Winkler, Stephen J. O’Brien, Jing Li, Kaiyin Xiao, Tao Peng

**Affiliations:** ^1^ Department of Hepatobiliary Surgery, The First Affiliated Hospital of Guangxi Medical University, Nanning, 530021, Guangxi Province, China; ^2^ Experimental Center, The First Affiliated Hospital of Guangxi Medical University, Nanning, 530021, Guangxi Province, China; ^3^ Department of Pathology, The First Affiliated Hospital of Guangxi Medical University, Nanning, 530021, Guangxi Province, China; ^4^ Department of General Surgery, West China Hospital of Sichuan University, Chengdu, 610041, Sichuan Province, China; ^5^ Laboratory of Genomic Diversity, National Cancer Institute, National Institutes of Health, Frederick, MD 21702-1201, USA; ^6^ Department of Physiology and Pathophysiology, College of Basic Medical Science, Peking University, Health Science Center, Beijing, 100191, China

**Keywords:** transcatheter arterial chemotherapy, hepatocellular carcinoma, TP53, p21^waf1/cip1^, oxidative stress

## Abstract

In this study, we aim to investigate oxidative stress in hepatocellular carcinoma (HCC) tissues in patients receiving preoperative transcatheter arterial chemotherapy (TAC) and its association with prognosis. A total of 89 HCC patients enrolled in this study, 39 received preoperative TAC 1 week before surgery (pTAC group) and 50 did not (non-pTAC group). All patients underwent hepatectomy and postoperative TAC and were followed up to 400 weeks. Samples of liver tissue without HCC and hepatitis (*n* = 15) served as normal controls. Cellular levels of 8-hydroxy-2′-deoxyguanosine (8-OHdG), TP53, and p21^waf1/cip1^ were measured in both cancer and surrounding tissues using an immunohistochemistry assay. Taken together, our data suggested that preoperative TAC might postpone postoperative HCC relapse within 1 year via suppression of tumor cells by induction of high levels of oxidative stress.

## INTRODUCTION

Hepatocellular carcinoma (HCC) is one of the most common malignancies in China [1]. Transcatheter arterial chemoembolization (TACE) is a palliative treatment for patients with unresectable intrahepatic tumors to obtain degradation and possible consequential resection [2]. We found that postoperative adjuvant TACE seems to decrease the short-term HCC recurrence rate among patients with risk factors such as multiple nodules > 5 cm or vascular invasion [3]. When applying transcatheter arterial chemotherapy (TAC) without embolization agents rather than TACE for the treatment of patients with HCC and main portal vein thrombosis(MPVT), TAC as an adjunctive therapy significantly improved survival in HCC patients[4]. The main difference between TACE and TAC is that embolization agents are not used in TAC. Embolization agents play an important role in preoperative TACE to reduce tumor size and induce tumor necrosis by blocking tumor feeding artery. But routine preoperative TACE for resectable HCC is not recommended because whether it improves surgical outcome remains controversial, furthermore, preoperative TACE may cause a distinct risk of a resectable HCC becoming unresectable, or increases risk of perioperative complications such as liver failure [5, 6]. In contrast, although preoperative TAC is safer than the TACE for resectable HCC patients, but whether it could improve surgical outcomes was not fully studied so far.

The anti-tumor effect of TAC is attributed to oxidative stress induced by anti-neoplastic agents [7] via generation of both high and low levels of reactive oxygen species (ROS) [8]. During cancer chemotherapy, lipid peroxidation induced by oxidative stress generates electrophilic aldehydes that can attack many cellular targets [7]. ROS such as hydrogen peroxide, superoxide anion, hydroxyl group and oxygen are byproducts of cell metabolism [9]. ROS damage tissues and cellular components including membranes, DNA, and proteins. Oxidative stress occurs when the ROS concentration exceeds the antioxidant capability of a cell or tissue [10]. *In vitro* experiments revealed that effects ROS can stimulate cell growth [11]. ROS regulate genes via protein kinase C activation, oxidative damage, and/or direct activation of transcription factors. The effects of ROS on gene transcription may also inhibit normal cell apoptosis via modulation of myc, bcl-2, and TP53 expression, resulting in an increase in cell number [12]. The most damaging species among ROS is the hydroxyl radical, which is responsible for base modifications such as the generation of 8-hydroxy-2′-deoxyguanosine (8-OHdG) [13], involving oxidation of guanine at the C-8 position [14]. 8-OHdG can induce G-C to T-A transversion during DNA replication, and is thus used as a marker of oxidative DNA damage [15].

*TP53* is known as a guardian gene and it is thought that TP53 loss is responsible for a lack of apoptotic signals in tumor cells and thus for their uncontrolled proliferation and recurrence [16]. Chemotherapeutic agents induce oxidative stress *in vivo* [17]. TP53-dependent and -independent pathways modulate the cytotoxic effects of common anti-neoplastic agents [18]. Moreover, TP53-dependent cycle arrest is primarily mediated by the cyclin-dependent kinase inhibitor p21^waf1 /cip1^ [19]. Mutant or absent TP53 status has been associated with resistance to radiation therapy and to apoptosis-inducing chemotherapy [20]. Mutations in the *TP53* gene have frequently been detected in patients with recurrent HCC, and the interval between resection and HCC recurrence was significantly later in patients with wild-type *TP53* than in those with mutations [21].

Chemotherapy could trigger DNA repair in HCC, raising the hypothesis that the overall efficacy of TAC on HCC depends on the cellular response to oxidative stress caused by chemotherapeutic agents in both cancerous and surrounding tissues. Therefore, we investigated oxidative stress in liver tissues in HCC patients receiving preoperative TAC to determine the effect of oxidative stress on changes in HCC tissue and its association with prognosis.

## RESULTS

### 8-OHdG levels in tumor tissues

**Figure 1:**
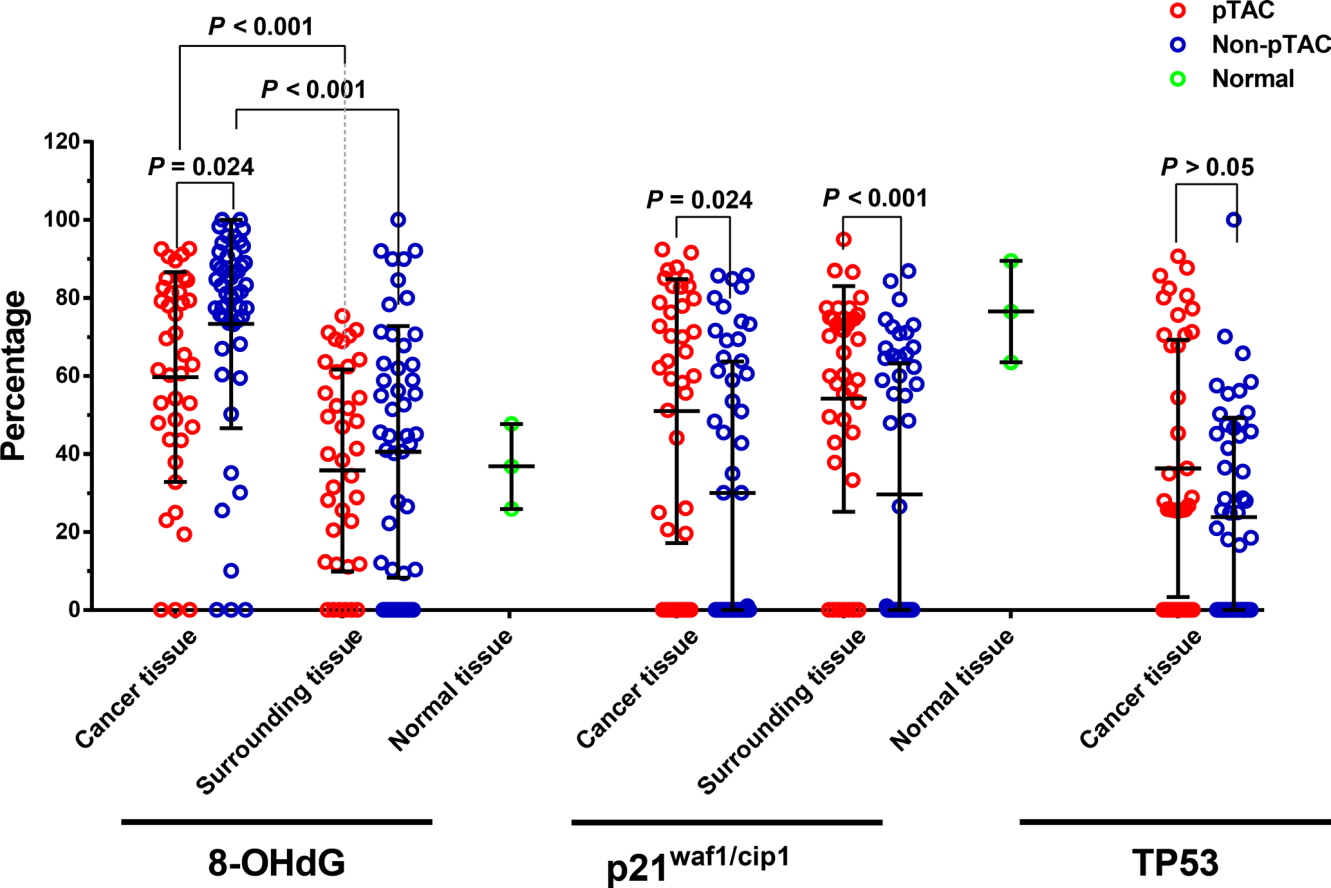
Comparison of 8-OHdG and p21^waf1/cip1^ levels in the pTAC, non-pTAC, and control groups (Mean ± SD)

8-OHdG levels in cancer tissues were higher in the pTAC (positive ratio 59.7 ± 26.9%) and non-pTAC groups (73.3 ± 26.7%) than in the control group (38.6 ± 33.1%; *F* = 9.516, *p* = 0.001). Of note, 8-OHdG levels in cancer tissues were lower in the pTAC group than in the non-pTAC group (F = 9.516, *p* =0.024). However, there was no significant difference in 8-OHdG levels among surrounding tissues in the pTAC (35.8 ± 25.9%), non-pTAC groups (40.5 ± 32.3%) and control tissues (*F* = 0.273, *p* = 0.762). In both the pTAC (*t* = 7.101, *p* < 0.001) and non-pTAC groups (*t* = 8.040, *p* < 0.001), 8-OHdG levels in cancer tissues were significantly higher than in surrounding tissues (Figure 1). In addition, 8-OHdG levels in cancer and surrounding tissues were closely associated in both the pTAC (*r* = 0.651, *p* < 0.001) and non-pTAC groups (*r* = 0.493, *p* = 0.001).

### TP53 expression in tumor tissues

TP53 expression levels in cancer tissues in the pTAC (31.9 ± 32.1%) and non-pTAC groups (29.2 ± 30.0%) were not significantly different (*t* = 0.41, *p* = 0.683). TP53 was not detected in surrounding or normal tissues.

### p21^waf1/cip1^ expression in tumor tissues

p21^waf1/cip1^ expression in cancer tissues was significantly higher in the pTAC group (51.0 ± 33.8%) than in the non-pTAC group (30.0 ± 33.7%; *F* = 13.459, *p* = 0.003), and in both cases was significantly lower than in the control group (76.5 ± 17.1%; *F* = 13.459, *p* < 0.001). No significant difference in p21^waf1/cip1^ expression was found between cancer and surrounding tissues (*p* > 0.05) in the pTAC and non-pTAC groups. However, p21^waf1/cip1^ expression in surrounding tissue was significantly higher in the pTAC group (54.1 ± 28.9%) than in the non-pTAC group (29.6 ± 33.5%; *p* < 0.001), and in both cases was significantly lower than in the control group (*F* = 16.613, *p* < 0.001; Figure 1). The association between p21^waf1/cip1^ levels in cancer and surrounding tissues was significant in the non-pTAC group (*r* = 0.872, *p* < 0.001) but not in the pTAC group (*r* = 0.04, *p* = 0.808).

### Alanine aminotransferase (ALT) and aspartate aminotransferase (AST)

**Figure 2:**
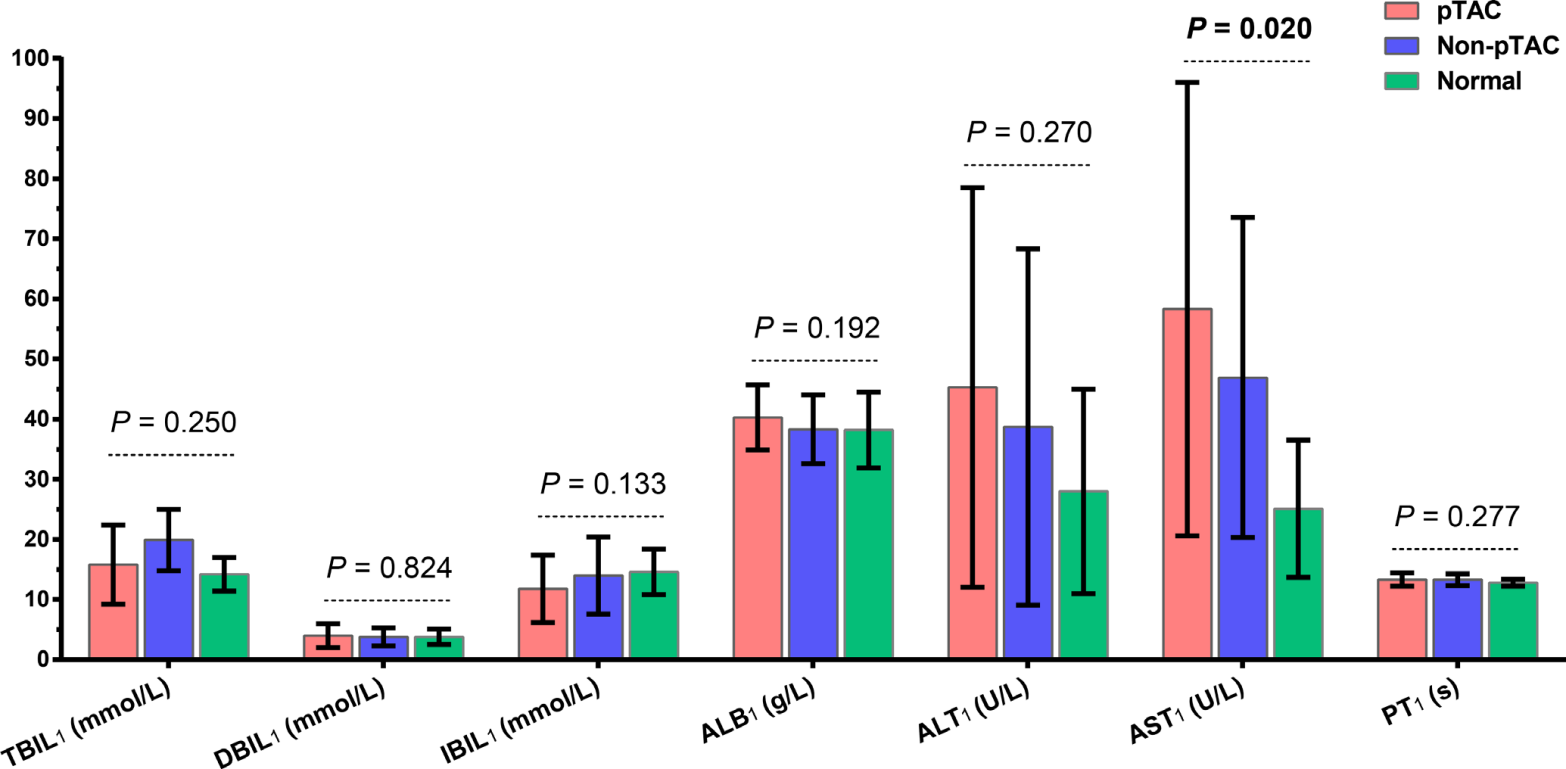
Liver function results on admission for patients in the pTAC, non-pTAC, and control groups *P* > 0.05 for AST_1_ in pTAC group versus non-pTAC group (Mean ± SD).

There was no significant difference in baseline liver function indices between pTAC and non-pTAC patients on admission (Figure 2). However, in the pTAC group, ALT and AST levels after TAC 3 days were significantly higher than at baseline (*Z* = –4.808, *p* < 0.001). At 1 week after surgery, there was no significant difference in ALT (*p* = 0.076) and AST(*p* = 0.427) levels or in the change in ALT (ΔALT; *p* = 0.676) and AST (ΔAST; *p* = 0.281) levels between the pTAC and non-pTAC groups. The 8-OHdG level in cancer tissue was associated with ΔAST (*r* = –0.348, *p* = 0.030) in the pTAC group. Similarly, 8-OHdG in cancer tissue was associated with ΔALT (*r* = –0.281, *p* = 0.048) in the non-pTAC group (Table 1).

**Table 1:** Association between ΔALT and ΔAST and 8-OHdG, p53, and p21^waf1/cip^^1^ in cancer tissues

Variables	8-OHdG		TP53		p21^waf1/cip1^	
	pTAC	Non-pTAC	pTAC	Non-pTAC	pTAC	Non-pTAC
ΔALT (ALT_2_-ALT_1_)	r = –0.180	r = –0.281*	r = –0.022	r = –0.042	r = –0.077	r = 0.015
ΔAST (AST_2_-AST_1_)	r = –0.348*	r = –0.176	r = 0.055	r = –0.008	r = –0.157	r = 0.050

Note: *Association is significant at the 0.05 level (2-tailed). ALT_1_ and AST_1_ were the preoperative levels of ALT and AST before hepatectomy. ALT_2_ and AST_2_ were the post-operative levels of ALT and AST after hepatectomy 1 week.

### Survival analysis

**Figure 3:**
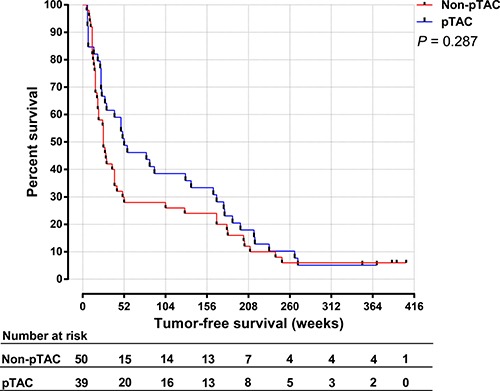
Tumor-free survival time for HCC patients in the pTAC and non-pTAC groups

**Figure 4:**
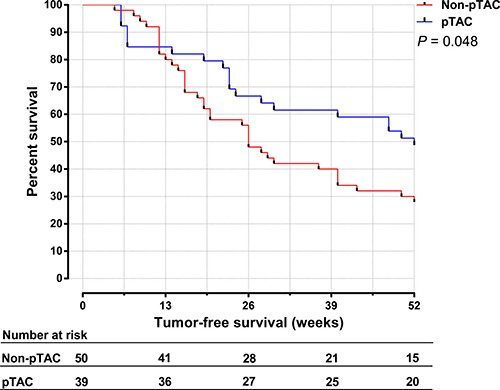
The 1-year cumulative tumor-free survival time for HCC patients in the pTAC and non-pTAC groups

**Figure 5:**
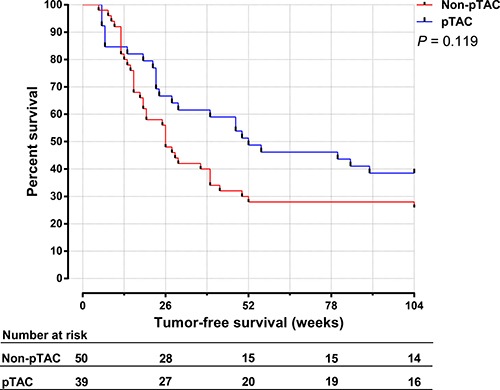
The 2-year cumulative tumor-free survival time for HCC patients in the pTAC and non-pTAC groups

The median tumor-free survival time was 52 weeks in the pTAC group and 26 weeks in the non-pTAC group, the 1-, 3-, 5-, 8-year recurrence rate was 51.3%, 66.7%, 89.7%, 94.9% respectively in the pTAC group and 72.0%, 76.0%, 94.0%, 94.0% respectively in the non-pTAC group, however the differences were not significant (*p* = 0.287). Figure 3 shows that between 52 and 156 weeks after surgery, the tumor-free survival rate was higher in the pTAC group (51.3%–33.3%) than in the non-pTAC group (30.0%–26.0%). The 1-year (< 52 weeks) cumulative tumor-free survival rate was significantly higher in the pTAC group (51.3%) than in the non-pTAC group (30.0%; *p* = 0.048; Figure 4), whereas the 2-year (< 104 weeks) cumulative tumor-free survival rate did not significantly differ between the groups (41.0% vs. 28%; *p* = 0.119; Figure 5). There was significant correlation between tumor-free survival time and tumor 8-OHdG levels in the pTAC group (*r* = –0.356, *p* = 0.026).

Univariate analysis shows that Barcelona Clinic Liver Cancer (BCLC) classification stage (*p* = 0.001), HBsAg positive (*p* = 0.003), AFP levels > 400 ng/ml (*p* = 0.033), tumor size (*p* = 0.002) and Vascular invasion (*p* = 0.028) were significantly influence tumor-free survival time (Table 2). We used a multivariate analysis, including the predictors associated with tumor-free survival time mentioned above, with the Cox proportional hazard method to evaluate the factors influencing tumor-free survival time. The analysis revealed that BCLC stage (*p* = 0.045) and vascular invasion (*p* = 0.010) were independent risk factors for the post-operative tumor-free survival time of HCC patients (Table 3).

**Table 2:** Associations between clinical factors and tumor-free survival

Variables	Patients (*n* = 89)	Tumor–free survival		
		MRT(weeks)	HR* (95% CI)	P*
Age (yrs)				
≤ 46	49	32	Ref.	
> 46	40	42	0.85 (0.76–1.02)	0.112
Gender				
Male	79	30	Ref.	
Female	10	43	0.69 (0.35–1.39)	0.302
Race				
Han	73	38	Ref.	
Minority	16	36	1.07 (0.91–1.45)	0.284
BMI				
≤ 25	61	39	Ref.	
> 25	28	32	1.02 (0.84–1.51)	0.475
Smoking status				
None	70	41	Ref.	
Ever	19	34	0.91 (0.88–1.38)	0.250
Drinking status				
None	60	40	Ref.	
Ever	29	30	0.69 (0.55–1.21)	0.391
Family history of liver ailments				
No	82	40	Ref.	
Yes	7	19	1.81 (0.82–3.97)	0.139
Child-Pugh score				
5	46	41	Ref.	
6	43	35	1.81 (0.84–2.65)	0.413
Adjuvant pTAC				
No	50	26	Ref.	
Yes	39	52	0.79 (0.51–1.22)	0.294
BCLC stage				
A	21	168	Ref.	**0.001**
B	63	26	2.58 (1.49–4.47)	**0.001**
C	5	12	4.46 (1.60–12.39)	**0.004**
Cirrhosis				
No	41	37	Ref.	
Yes	48	30	1.44 (0.93–2.23)	0.104
HBsAg				
Negative	15	136	Ref.	
Positive	74	26	2.64 (1.38–5.05)	**0.003**
Anti-HBV postoperatively				
No	17	52	Ref.	
Yes	72	30	0.89 (0.52–1.53)	0.674
AFP (ng/ml)				
≤ 400	40	40	Ref.	
> 400	49	28	1.62 (1.04–2.52)	**0.033**
PT1 (s)				
≤ 13	37	43	Ref.	
> 13	52	26	1.36 (0.88–2.11)	0.169
Pathological grade				
Well	66	30	Ref.	0.963
Moderately	10	50	0.97 (0.49–1.89)	0.919
Poorly	13	40	0.92 (0.50–1.71)	0.788
Oncological behavior Tumor size				
≤ 5 cm	28	128	Ref.	
> 5 cm	61	26	2.17 (1.34–3.51)	**0.002**
No. of tumors				
Single (*n* ***=***1)	65	48	Ref.	
Multiple (*n*≥1)	24	25	1.58 (0.97–2.57)	0.066
Vascular invasion				
Absence	84	50	Ref.	
Presence	5	19	2.27 (1.36–3.79)	**0.028**
Capsule				
Absence	74	30	Ref.	
Presence	15	43	1.02 (0.57–1.81)	0.951

Note: *HR and *P* value for univariate survival analysis.

**Table 3:** Factors influencing tumor-free survival time (Cox regression)

Variables	B	SE	Wald	df	P	HR	95.0% CI HR	
							Lower	Upper
HBsAg	0.226	0.423	0.285	1	0.594	1.253	0.547	2.870
AFP levels	0.138	0.278	0.249	1	0.618	1.148	0.667	1.979
Tumor size	0.461	0.461	1.002	1	0.371	1.586	0.643	3.916
BCLC stage	0.583	0.290	4.030	1	**0.045**	1.791	1.014	3.166
Vascular invasion	1.094	0.321	11.619	1	**0.010**	2.988	1.023	3.343

Abbreviations: HR, hazard ratio; 95% CI, 95% confidence interval;

## DISCUSSION

Our study showed that regardless of whether HCC patients received preoperative TAC or not, 8-OHdG levels were significantly higher in tumor tissues than in control tissues. This indicates an increase in oxidative stress in cancer tissues compared to normal liver tissues. Moreover, 8-OHdG levels were higher in cancer tissues than in surrounding tissues in the pTAC and non-pTAC groups, similar to findings in lung cancer [22]. renal cell carcinoma [23], and carcinoma of the large intestine [24], suggesting higher levels of oxidative DNA damage in cancer cells than in surrounding tissues. This supports that increased 8-OHdG levels were associated with a high risk of hepatocarcinogenesis [25]. Some findings not only indicate the active proliferation and high oxygen metabolism in cancer tissues but also probably reflect the inflammation that occurs in tumor tissues [14, 26, 27]. 8-OHdG levels are also likely to reflect cell differentiation, proliferation, and invasion in HCC [28]. We found that 8-OHdG levels in HCC tissues were significantly lower in the pTAC group than in the non-pTAC group, probably because the active metabolism and chemoresponsiveness of HCC cells were inhibited by the antitumor pTAC drugs. However, residual tumor cells might escape from pTAC in a fortified oxidative stress repair mechanism. At the same time, liver aid used to protect patients after TAC may need to be taken into consideration. In peritumoral tissues, there was no difference in 8-OHdG between the pTAC and non-pTAC groups, indicating that the effects of TAC differs between tumor and surrounding tissues: TAC can decrease 8-OHdG levels in cancer tissues, but has no effect on 8-OHdG in peritumoral tissue in HCC. After chemotherapy, a significant decrease in urinary 8-OHdG was found in small–cell lung cancer patients with a complete or partial remission response, which reflects a decrease in tumor mass and thus relief of oxidative stress; by contrast, a significant increase in urinary 8-OHdG was observed after chemotherapy in patients with no response or with progressive disease, and patients with no response to radiotherapy had higher 8-OHdG levels than patients with a response [29]. Toyokuni et al. [30] found that high antioxidant levels induced by persistent oxidative stress increases the chemotherapy resistance of cancer cells. During cancer chemotherapy, lipid peroxidation induced by oxidative stress generates numerous electrophilic aldehydes that can attack many cellular targets. These oxidative products can slow the cell cycle progression of cancer cells and cause cell cycle checkpoint arrest that may interfere with the ability of anticancer drugs to kill cancer cells. The aldehydes may also inhibit drug-induced apoptosis by activating death receptors and inhibiting caspase activity [7]. These effects would also diminish the efficacy of treatment [7]. Accordingly, the 8-OHdG level in cancer tissues may remain high after TAC, suggesting that HCC cells may be resistant to a pro-chemotherapy program. This could provide a useful hint for clinicians considering a change in chemotherapy program and for detection of early recurrence after tumor resection.

ROS as signaling molecules can mediate apoptosis via a TP53-dependent pathway. On encountering a DNA defect, TP53 protein encoded by wild-type *TP53* immediately stops the cell cycle and starts the DNA repair mechanism; if DNA repair fails, TP53 initiates apoptosis instead. The mechanism by which TP53 inhibits cell cycle progression is likely to involve activation of p21^waf1/cip1^ expression via transcription. The half-life of wild-type TP53 is too short to examine, and mutant TP53 can only be detected by immunohistochemistry. We found that TP53 expression in tumor tissues did not differ between the pTAC and non-pTAC groups, perhaps indicating the presence of cancer cells with mutant TP53 resistance to the TAC program used. This supports the view that tumor cells with mutant TP53 have greater potential for malignancy and invasion [31, 32]. The decrease in oxidative stress levels in cancer tissues in the pTAC group can probably be attributed to a repair mechanism mediated by a TP53-independent pathway. Ahn et al. [33] found that HCC occurrence and development were correlated with downregulation of p21^waf1/cip1^ protein expression. In our study, p21^waf1/cip1^ protein expression was highest in the control tissues, was lower in peritumoral than in control tissues, and was lowest in cancer tissues. This is in agreement with observations by Mise et al. [34], but not with results reported for HCC, hepatic cirrhosis, and normal hepatic tissues by Zhang et al [35]. We do not know whether this contradiction can be attributed to incompetence p21^waf1/cip1^ protein expression in tumor tissues or not [36]. In the pTAC group, p21^waf1/cip1^ protein expression increased in cancer tissues because of repair of oxidative DNA damage in cells induced by chemotherapy. We found that although TP53 protein levels in cancer tissues were similar in both HCC groups, p21^waf1/cip1^ expression was significantly higher in the pTAC group than in the non-pTAC group, indicating that in spite of defective TP53 protein in tumor cells, oxidative stress was upregulated in response to chemotherapy via an alternative pathway. p21^waf1/cip1^ expression was significantly lower in peritumoral tissues in both the pTAC and non-pTAC groups than in control tissues. It is likely that this is related to inadequate wild-type TP53 function in peritumoral tissues in HCC. Peritumoral p21^waf1/cip1^ expression levels were higher in the pTAC group than in the non-pTAC group, so it is possible that p21^waf1/cip1^ upregulation involved a TP53-independent pathway or that hOGG1 levels increased after TAC.

Anti-neoplastic agents used in TACE not only cause necrosis of tumor cells but also induce liver dysfunction [37]. Our data revealed that in the pTAC and non-pTAC groups, there was no significant difference in ALT and AST levels between preoperative and post surgery time point. At 3 days after TAC administration, ALT and AST levels were significantly higher than on admission, suggesting that after chemotherapy, patients sensitive to TAC suffer from damage not only to the liver but also to other organs [38]. Moreover, we found that ΔAST was negatively associated with 8-OHdG in pTAC group, whereas ΔALT was negatively associated with 8-OHdG in the non-pTAC group (Table 1), although these correlations were mild, indicating that the levels of oxidative stress in cancer tissues might affect the liver function.

Survival analysis (Figure 3) showed that the tumor-free survival time did not significantly differ between the pTAC and non-pTAC groups, similar to findings by Kaibori et al. [39]. The median tumor-free survival time was longer in the pTAC group (52 weeks) than in the non-pTAC group (26 weeks), similar to results reported by Choi et al. [40]. 8-OHdG levels have been used as an independent prognostic factor for HCC recurrence, and patients with greater intrahepatic oxidative stress had a higher incidence of HCC recurrence [41, 42]. It is interesting to find that 8-OHdG levels in cancer tissues were significantly lower in the pTAC group than in the non-pTAC group. If overlook the interval from pTAC to surgical resection, this might suggest that HCC cells which metabolic active and sensitive to chemotherapy were attenuated by the antitumor drugs used, although it is also possible that the number of cases in our study was not much enough to properly reflect differences in overall survival time. We found that the 1-year cumulative tumor-free survival rate was significantly higher in the pTAC group than in the non-pTAC group. However, the 2-year cumulative tumor-free survival rate was not significantly different between the two groups. These results indicate that preoperative TAC can reduce the HCC recurrence risk in the short term after radical hepatectomy (1 year). Thus, a multimodal therapy strategy to prevent HCC recurrence within the first year after surgery seems to be warranted.

In the pTAC group, tumor-free survival time was inversely correlated with 8-OHdG levels in cancer tissues. This suggests that the levels of 8-OHdG generated by residual tumor cells that escape chemotherapy may reflect the prognosis of HCC patients undergoing preoperative TAC. Other researchers found that high oxidative stress in non-cancerous liver tissue from HCC patients was associated with a higher incidence of HCC recurrence [41, 42]. However, we did not find any correlation between 8-OHdG levels in surrounding tissues and survival time. This may be because the population in Guangxi province is exposed to high levels of aflatoxin B_1_ (AFB_1_) [43]. Liver damage caused by hepatitis viruses and AFB_1_ may generate more 8-OHdG, which might influence the survival time of HCC patients in Guangxi province.

We found that BCLC stage of HCC, vascular invasion influenced HCC recurrence. This is consistent with previous observations [41] and supports the view that the greater the tumor malignancy and invasion (high pathologic staging and grading), the poorer is the survival rate.

One limitation of this study is the small number. It is because we had applied strict criteria of enrollment. We included only HCCs received radical resection and excluded those received variable dosage of TACE to control the variations. A larger sample size in the future might strengthen the findings in this study. Another concern of bias came from the post-resection TAC/TACE that all our HCCs received. Whether HCC patients could be beneficial from post-resection TAC/TACE remains controversial [44, 45]. So when retrospectively studying this population 10-yrs ago, we recruited only HCCs received postoperative TAC at 1, 3, and 6 months after hepatectomy to ensures comparable baseline between the pTAC and non-pTAC groups. Finally, in this study we tried to ensure the patients’ clinical baselines in two groups were comparable. It is ideal to get liver tissues before and after TAC therapy and compare their oxidative status. But in practice it is hard to get approval from HCC patients to do biopsy (to get liver tissues) before TAC, especially when the tumors were resectable.

In conclusion, this study clearly showed that after preoperative TAC, the status of oxidative stress in cancer tissue changed in HCC. Oxidative stress levels were higher in HCC tissues than in surrounding tissues and normal liver tissue. Hepatic cancer cells probably escape intervention chemotherapy via a fortified oxidative stress repair mechanism. Differences in the expression of DNA damage biomarkers and cell-cycle regulators in HCC and surrounding tissues suggest that a distinct cellular response to oxidative challenge might have clinical implications. Preoperative TAC postponed early HCC relapse after hepatectomy, for which the mechanism might involve lower levels of oxidative stress in the tumor microenvironment.

## MATERIALS AND METHODS

### Subject recruitment and sample collection

Between April 2006 and April 2008, a total of 463 HCC patients were hospitalized in the Hepatobiliary Surgery Department of First Affiliated Hospital of Guangxi Medical University. Of these, 224 (48.4%) were selected for surgery (including liver transplantation) and 239 (51.6%) received either TACE (*n* = 180) for unresectable tumors or percutaneous ablation therapy (PAT; *n* = 59) for single HCC tumors < 2 cm in diameter. Of the 224 surgical patients, 89 (39.7%) underwent radical hepatectomy (cutting-edge to tumor boundary > 2 cm and/or no tumor visible at the cutting-edge under a microscope.) and were divided into two categories: 39 cases who received preoperative TAC (pTAC group) and 50 who did not (non-pTAC group). The 39 pTAC patients underwent transcatheter arterial angiography to differentiate tumor masses and cirrhotic nodules (lipiodol was not used) before TAC administration. A fixed regime (pirarubicin 50 mg + cisplatin 50 mg + fluorouracil 1 g) was used for all TAC patients. Lipiodol was not used for embolization in these patients because it may cause tumor necrosis. None of the 89 enrolled HCCs received previous HCC treatment or antiviral treatment before preoperative TAC, none of them received Sorafenib before or after surgical/TACE intervention. And none of other HCCs received radiotherapy regardless its PVTT status. 101 HCC patients who received preoperative TAC with different drug doses were excluded. Liver function indices were measured before and on the 3rd day after TAC. Hepatectomy for pTAC patients was performed 1 week after the chemotherapy, and both the pTAC and non-pTAC groups received postoperative TAC at 1, 3, and 6 months after hepatectomy. Samples of 15 non-HCC liver tissues were obtained during operations for hemangioma, liver harvesting for transplantation, or accidental hepatorrhexis, and served as the control group. All control subjects were negative for hepatitis B (HBV) and hepatitis C virus (HCV) sero-markers. The type of hepatectomy for HCC depended on the location of tumor(s), the severity of concomitant cirrhosis, and liver function reserve. In all cases a surgical margin > 2 cm was achieved. The duration of Pringle's maneuver was not significantly different among the three groups. Demographic data for the 104 cases are summarized in Table 4. Formalin-fixed, paraffin-embedded specimens were collected and independently diagnosed by two pathologists (Z.-L. Lu, M.-Y. Wei). All patients were followed up via mail, telephone, or outpatient visit from 1 month after hepatectomy to December 31, 2015. The follow-up time was up to 406 weeks, and there was no patient drop-off. Informed consent was obtained from all participants. This study was approved by the institutional review board of Guangxi Medical University.

**Table 4:** Demographic data for patients in the pTAC, non-pTAC, and control groups

Variables	pTAC	Non-pTAC	Control	*p* value
patients	39	50	15	
Age (years)	46.8 (24–70)	45.5 (25–68)	45.1 (22–67)	0. 732
Gender				0.195
Male	37	42	10	
Female	2	8	5	
BMI	24.8	24.9	25.2	0.883
Race				0.246
Minority	4	12	3	
Han	35	38	12	
Smoking status				0.349
None	11	8	4	
Ever	28	42	11	
Drinking status				0.267
None	14	15	2	
Ever	25	35	13	
Family history of liver ailments				**< 0.001^4^**
Present	3	4	0	
Absent	36	46	15	
HBsAg				**< 0.001^4^**
Positive	35	39	0	
Negative	4	11	15	
Anti-HBV postoperatively	8	9	0	0.822
AFP				**< 0.001^4^**
> 400 ng/ml	22	27	0	
≤ 400 ng/ml	17	23	15	
Cirrhosis				**0.001^4^**
Present	24	24	1	
Absent	15	26	14	
Differentiation				0.311
Well	26	40	-	
Moderately	5	5	-	
Poorly	8	5	-	
Child-Pugh score	5.6 ± 0.9	5.2 ± 0.7	5.4 ± 0.7	0.869
Tumor size^1^				0.474
> 5 cm	29	32	9	
≤ 5 cm	10	18	6	
Portal vein invasion^2^				0.810
vp0	37	47	-	
vp1	1	2	-	
vp2	1	1	-	
vp3	0	0	-	
vp4	0	0	-	
BCLC stage^3^				0.679
0/A	9	12	-	
B	28	35	-	
C	2	3	-	

Note:

1. Tumor size: diameter for a single nodule, or sum of the diameter of each nodule for multiple tumors.

2. Portal vein tumor thrombosis based on the Iwao Ikai classification.[46]

3. BCLC stage: Barcelona Clinic Liver Cancer classification stage.

4. All *P* between pTAC versus non-pTAC group were > 0.05, which were not showed in table.

### Immunohistochemical assays for 8-OHdG, TP53, and p21^waf1/cip1^

**Figure 6:**
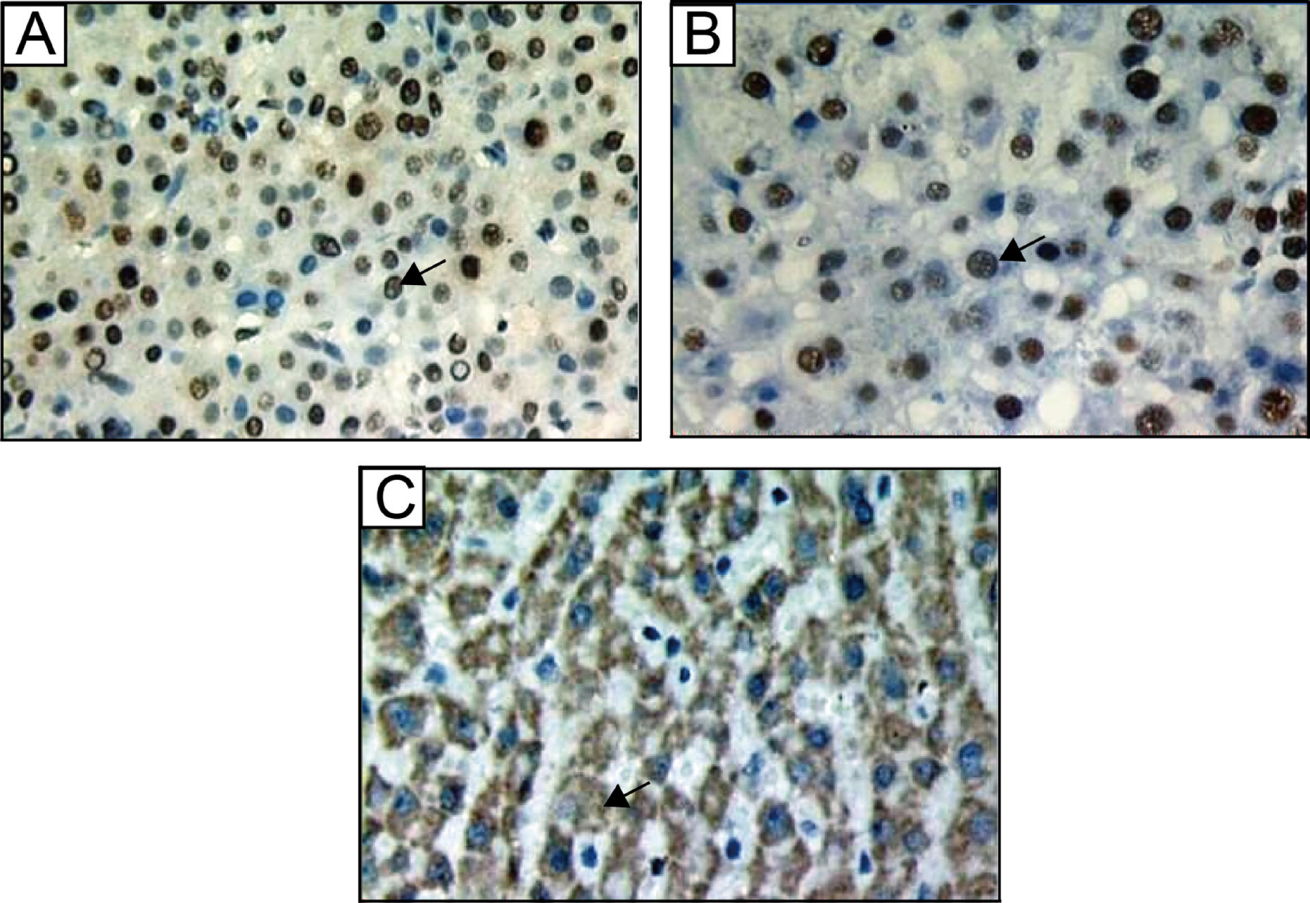
**Immunohistochemistry staining for (A) 8-OHdG, (B) p53, and (C) p21^waf1/cip1^ in liver tissues. Brown-yellow particles in (A, B) the nucleus of hepatic cells or (C) the cytoplasm indicate positivity. Magnification: 400×, arrow is positive expression**.

Tissues were fixed in formalin (10%) immediately after resection, dehydrated in absolute ethanol and embedded in paraffin. Serial sections (5 μm) were prepared for immunohistochemical analysis. The 8-OHdG, TP53 and p21^waf1/cip1^ levels were measured by hepatic immunohistochemical staining. Using monoclonal antibodies for 8-OHdG (N45.1; Japanese Aged Control Institute, Japan), TP53 and p21^waf1/cip1^ (mouse anti-human; Maixin-Bio Fuzhou, China). For 8-OHdG immunostaining, tissue sections were pretreated with 100 μg/ml ribonuclease A in phosphate buffer saline (PBS) in order to inhibit nonspecific binding to RNA. Immunohistochemistry was performed using a modified streptavidin- biotinylated peroxidase technique using Ultra Sensitive TM S-P or EliVision TM plus Kit from Fuzhou Maixin. Nonspecific protein staining was blocked by rabbit or goat serum for 30 min at room temperature. The sections were incubated with primary antibody at 4°C overnight. The sections were rinsed with PBS and incubated with biotinylated secondary antibodies for 30 min. They were then washed and treated with 0.3% hydrogen peroxide in methanol for 30 min in order to inhibit the activity of any endogenous peroxide. The slides were washed, incubated with streptavidin-biotin-peroxidase complex for 30 min, and developed according to the manufacturer's directions. The sections were subsequently counterstained with DAB. Brown-yellow staining in cell nuclei was observed for 8-OHdG- and TP53-positive cells (Figure 6A, 6B) and in cytoplasm for p21^waf1/ci^p1-positive cells (Figure 6C). The diagnosis was made by two pathologists independently, and the number of positive nuclei (p21^waf1/cip1^-was positive cytoplasm) in four to five randomly selected fields (400×) were counted and expressed as the ratio of positive nuclei to total nuclei (p21^waf1/cip1^-was the ratio of positive cytoplasm to total cytoplasm).

### Statistical analysis

Data for continuous variables are expressed as mean ± SD. One-way ANOVA and Fisher's least significant difference (for multiple comparisons) were used to evaluate differences in 8-OHdG and p21^waf1/cip1^ levels in liver tissues. A × test was used to examine the ratio of gender, race, smoking, and drinking in different categories. The Wilcoxon rank test was performed to assess changes in liver function. A paired *t*-test was used to test differences in 8-OHdG, TP53, and p21^waf1/cip1^ levels in cancer and surrounding tissues. The Spearman rank correlation coefficient was used to test associations. The Kaplan–Meier method was used to graphically depict the tumor-free survival time for HCC patients, and the log-rank test was applied to compare the median tumor-free survival time between the pTAC and non-pTAC groups. A Cox proportional hazards model was used to evaluate factors influencing tumor-free survival time. A two-tailed *p* value <0.05 was considered statistically significant.

## Abbreviations

HCC: hepatocellular carcinoma
TAC: transcatheter arterial chemotherapy
pTAC: preoperative transcatheter arterial chemotherapy
TACE: transcatheter arterial chemoembolization
ROS: reactive oxygen species
HR: hazard ratio
95% CI: 95% confidence interval
Ref.: reference
8-OHdG: 8-hydroxy-2′-deoxyguanosine.
